# Enhancing Visuospatial Working Memory Performance Using Intermittent Theta-Burst Stimulation Over the Right Dorsolateral Prefrontal Cortex

**DOI:** 10.3389/fnhum.2022.752519

**Published:** 2022-03-17

**Authors:** Ronald Ngetich, Donggang Jin, Wenjuan Li, Bian Song, Junjun Zhang, Zhenlan Jin, Ling Li

**Affiliations:** Ministry of Education (MOE) Key Lab for Neuroinformation, High-Field Magnetic Resonance Brain Imaging Key Laboratory of Sichuan Province, Center for Psychiatry and Psychology, School of Life Sciences and Technology, University of Electronic Science and Technology of China, Chengdu, China

**Keywords:** working memory, intermittent theta-burst stimulation (iTBS), right dorsolateral prefrontal cortex (rDLPFC), n-back task, neuroplasticity

## Abstract

Noninvasive brain stimulation provides a promising approach for the treatment of neuropsychiatric conditions. Despite the increasing research on the facilitatory effects of this kind of stimulation on the cognitive processes, the majority of the studies have used the standard stimulation approaches such as the transcranial direct current stimulation and the conventional repetitive transcranial magnetic stimulation (rTMS) which seem to be limited in robustness and the duration of the transient effects. However, a recent specialized type of rTMS, theta-burst stimulation (TBS), patterned to mimic the natural cross-frequency coupling of the human brain, may induce robust and longer-lasting effects on cortical activity. Here, we aimed to investigate the effects of the intermittent TBS (iTBS), a facilitatory form of TBS, over the right DLPFC (rDLPFC), a brain area implicated in higher-order cognitive processes, on visuospatial working memory (VSWM) performance. Therefore, iTBS was applied over either the rDLPFC or the vertex of 24 healthy participants, in two separate sessions. We assessed VSWM performance using 2-back and 4-back visuospatial tasks before iTBS (at the baseline (BL), and after the iTBS. Our results indicate that the iTBS over the rDLPFC significantly enhanced VSWM performance in the 2-back task, as measured by the discriminability index and the reaction time. However, the 4-back task performance was not significantly modulated by iTBS. These findings demonstrate that the rDLPFC plays a critical role in VSWM and that iTBS is a safe and effective approach for investigating the causal role of the specific brain areas.

## Introduction

Working memory (WM) is a daily used and highly researched cognitive domain. In essence, there is a considerably high demand for WM in complex cognitive task processing, but yet it remains a very limited resource (Baddeley, 2003; Luck and Vogel, 2013; Cowan, 2014; Bruning and Lewis-Peacock, 2020). To contextualize this, we need to remember the questions as we actively endeavor to answer them, we also need to remember patterns and sequences of events to do maths. Therefore, it is arguable that WM is necessary for us to carry out complex cognitive processes such as problem-solving and decision making. WM has been defined as a limited-capacity cognitive system that involves actively but transiently maintaining and manipulating goal-relevant information (Baddeley, 2010; Cowan, 2014; Wang et al., 2019). While WM is considered a pivot for cognitive processing, the dorsolateral prefrontal cortex (DLPFC) is a major region involved in the regulation of crucial cognitive processes ranging from WM, attention, cognitive control, to decision making (Goldman-Rakic, 1995; Cieslik et al., 2013; Taren et al., 2017).

The modulation of working memory (WM) performance has been consistently used in the study and intervention of psychiatric conditions such as schizophrenia and major depressive disorders (Oliveira et al., 2013; Hoy et al., 2016; Gärtner et al., 2018). Whereby, stimulation such as the intermittent theta-burst stimulation (iTBS) and continuous theta-burst stimulation (cTBS) have been applied often over the implicated WM brain areas including the dorsolateral prefrontal cortex (DLPFC), to enhance or inhibit their neural activity, respectively (Plewnia et al., 2014; Chistyakov et al., 2015; Cheng et al., 2016). We, therefore, targeted DLPFC for enhancement using iTBS. Specifically, we stimulated the right middle frontal gyrus (MFG), corresponding to the rDLPFC. Importantly, the findings of the previous studies indicate a possible hemispheric specialization in the processing of the verbal and visuospatial content. In essence, the processing of the visuospatial information has been primarily linked to the right hemisphere, and this also applies to the VSWM (Jonides et al., 1993; Kessels et al., 2000, 2002). In addition, a previous fMRI study found increased activation in the right ventrolateral and frontopolar prefrontal cortex during the performance of the spatial WM task (Manoach et al., 2004). On the other hand, a recent study applying lower frequency rTMS over the right DLPFC found a deterioration in visual working memory (VWM) (Fried et al., 2014). Furthermore, a previous meta-analytic study observed task-specific activations in the prefrontal cortex (PFC), with the verbal content associated with increased activation in the left PFC, whereas the visuospatial material was linked with increased activation in the right PFC (Owen et al., 2005). These converging evidence indicate the laterality of PFC in verbal WM and VSWM, with an indication of specialization of the left PFC in verbal WM and the right PFC in VSWM.

Moreover, previous studies suggest that applying transcranial magnetic stimulation over the DLPFC affects WM performance. For instance, (Oliveri et al., 2001) found that applying single-pulse TMS over bilateral DLPFC disrupts visual-object and VSWM task performance. Another study found an enhancement in verbal digit span and visuospatial 2-back task when high-frequency repetitive TMS (rTMS) was applied over the left DLPFC (Bagherzadeh et al., 2016). Furthermore, the extant literature suggests that continuous theta-burst stimulation (cTBS) over the left DLPFC decreases verbal WM task performance (Schicktanz et al., 2015; Vékony et al., 2018). While, our previous study using the visuospatial n-back task, found that applying cTBS over the right DLPFC impairs performance in a 2-back task (Ngetich et al., 2021). On the other hand, a study using the verbal WM n-back task indicates that iTBS over the left DLPFC enhances working memory performance (Hoy et al., 2015). Taken together, these studies underscore the importance of DLPFC in WM and the effectiveness of TMS in neuromodulation.

To assess the impact of iTBS on VSWM, we administered pre-and post-stimulation 2-back and 4-back VSWM tasks and measured the effect of stimulation based on the d prime (d’) scores and the reaction time (RT). We chose iTBS because of its potentiation effect (Huang et al., 2005; Chung et al., 2018a). An outstanding question, however, is how does iTBS modulate VSWM performance? Here, we aimed to establish whether a similar enhancement effect as that reported in Hoy et al. (2015) could be observed in a visuospatial n-back task following iTBS over the rDLPFC. The findings of the present study would greatly contribute to the growing literature on the cognitive effects of TBS over the focal brain areas. Additionally, the evidence that iTBS is efficacious as a treatment complement to pharmacotherapy in refractory neuropsychiatric disorders such as depression (Plewnia et al., 2014; Cheng et al., 2016; Ngetich et al., 2021), makes this study even more crucial.

It is worth noting that theta-burst stimulation (TBS) is a variant of TMS that uses gamma frequency trains applied in the rhythm of theta (thus mimicking theta-gamma coupling involved in the working and the long-term memory processes) (Chung et al., 2018b; Ngetich et al., 2020). The original study by Huang et al. (2005) indicates that TBS consist of a 50 Hz triplet of pulses interspersed at 5 Hz (repeated every 200 ms), and categorizes TBS into three types based on their stimulation patterns. The first type, which has also been applied in the present study is the iTBS. Under this paradigm, a 2 s TBS train is repeated every 10 s for 190 s to obtain a total of 600 pulses per session. The second type is cTBS, which involves a 40 s sustained application of the TBS train (600 pulses). Finally, the third type, intermediate theta-burst stimulation (imTBS) consists of a 5 s TBS train repeated every 15 s for 110 s to yield 600 pulses per session. According to the aforementioned study which is based on the motor cortex, iTBS led to increased motor evoked potential (MEP), while cTBS decreased the MEP, with no significant effect on MEP after imTBS of the motor cortex (Huang et al., 2005). These findings have considerably influenced the neuromodulation studies and intervention, with TBS currently used to investigate the functional roles of brain areas beyond the motor cortex, and importantly, it has been incorporated into the therapeutic approaches for psychiatric conditions.

Furthermore, the various TBS paradigms, especially iTBS and cTBS are associated with varied effects on neuronal activity. It should be noted that the mammalian brain consists of intricately interconnected neurons and synapses. This intricate but flexible neuronal network can be regulated by the plastic nature of the inter-neuron synaptic transmissions (Li et al., 2019). Importantly, the two main long-term manifestations of synaptic plasticity, long-term potentiation (LTP), and long-term depression (LTD) are instigated by postsynaptic Ca^2+^ changes in concentration (Blitzer et al., 2005). Therefore, it is anticipated that iTBS, which consists of short intermittent trains of bursts, results in excitation related to a temporary influx of Ca^2+^ and leads to an LTP-like effect. Conversely, cTBS train application facilitates an intensified inter-neuron depression effect through a sustained influx of Ca^2+^, and in the process, it overpowers the excitatory impact and causes an LTD-like effect (Huang et al., 2011).

However, despite its efficacy in the treatment of neuropsychiatric conditions, a small number of healthy-participant studies including Chung et al. (2018b) have found no significant behavioral impact of iTBS when applied over DLPFC. Interestingly, previous studies have found mixed results, where some studies suggest both iTBS related WM behavioral enhancement alongside the neurophysiological modulation of implicated brain areas (altered inter-regional connectivity) (Hoy et al., 2015). While some studies only demonstrate the neurophysiological effect of iTBS with no significant effect on behavioral performance (Chung et al., 2018b). More studies are thus required to verify whether the iTBS cortical modulation necessarily potentiates behavioral performance. Since most studies indicate that iTBS upregulate cortical activity (Wischnewski and Schutter, 2015; Chung et al., 2017, 2018a,b; Lowe et al., 2018) and DLPFC is implicated in WM (Rottschy et al., 2012; Hoy et al., 2015; Vékony et al., 2018; Ngetich et al., 2021), the present study sought to establish the behavioral impact of iTBS over the rDLPFC on VSWM.

## Materials and Methods

### Participants

Participants were recruited from the undergraduate students of the University of Science and Technology of China (UESTC). To be included in the study, the participants had to be healthy and right-handed [handedness was assessed using Edinburgh Handedness Inventory (Oldfield, 1971)], with normal or corrected to normal eyesight. Exclusion criteria included neurological or psychological illness or history of neuropsychiatric disorders, drug and substance abuse, left-handedness, inability to give informed consent, and having brain ferromagnetic implants. A total of 26 subjects were recruited. However, 2 participants had incomplete data as they could not attend all the sessions due to personal reasons, therefore, they were excluded from the experiment. Ultimately, the data of 24 participants (15 males, *M*_*age*_ = 22.25, *SD*_age_ = 1.6) were included in our analysis. All the experimental procedures adhered to the declaration of Helsinki and were approved by the ethics board of the UESTC.

### Procedure

**FIGURE 1:**
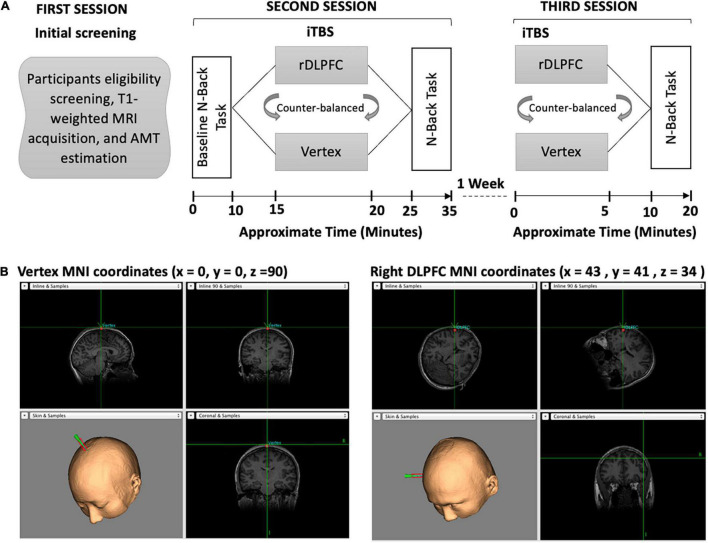
**(A)** Overview of the experimental paradigm. Our experiment consisted of three separate sessions. During the first session, the participants were screened on eligibility, had their T1-weighted images acquired, and AMT estimated. In the second session, participants performed the baseline n-back task followed by iTBS and then the post-stimulation n-back task. In the third session, the participants received iTBS and performed the post-stimulation n-back task. **(B)** The iTBS target brain areas, the vertex and the Rdlpfc, respectively.

The experiment consisted of 3 sessions, with the second and third sessions separated by a wash-out period of 7 days between them as described in Figure 1. All the participants attended all sessions. During the first session, the participants were screened on their eligibility, had their T1-weighted MRI images acquired, and active motor threshold (AMT) estimated. In the second session, the participants performed the baseline (BL) n-back task before receiving iTBS over either the vertex or the rDLPFC. Following the stimulation, the participants performed an n-back task. The third session was conducted a week after the second session, during which the participants received stimulation over the alternate site to that of the second session, and thereafter performed n-back task. In both stimulation sessions (second and third), iTBS was followed by a 5 min break before the behavioral task performance which lasted for approximately 10 min. The order of stimulation was counterbalanced between the subjects. Also, the order of *n*-values for the task was different for each session, i.e., session two could be 4-2-4-2-4-2 and session three could be 2-4-2-4-2-4 but was the same for each participant. The visuospatial n-back task experiment was designed using the E-Prime 2.0 (Psychology Software Tools, Pittsburgh, PA, United States). It consisted of the blue square presented on a black background computer screen with a resolution of 1,024 × 768 and a refresh rate of 60 Hz, at eight different random positions as shown in Figure 1.

Moreover, all participants received the same intensity of iTBS, that is, a uniform stimulation of 40% of the maximal machine output (MSO) was administered to either the rDLPFC or the vertex. For this reason, the purpose of measuring the AMT of each participant was to ensure that 40% of MSO did not surpass the individual’s stimulation tolerance level and hence enhanced participants’ safety. Besides, the application of uniform stimulation intensity is consistent with the previous studies including Ott et al. (2011); Vékony et al. (2018), and our recent cTBS study (Ngetich et al., 2021). All the participants gave written informed consent before they began the experiment and were given monetary compensation at the end of the experiment.

### Theta-Burst Stimulation and Neuronavigation

The participants received iTBS to either the rDLPFC or vertex in 2 separate sessions, with the site of stimulation counterbalanced across all the participants. This means 12 participants received stimulation over rDLPFC, and the other half received stimulation over the vertex in the second session. In the third session, participants who were stimulated over the rDLPFC in the second session, received vertex iTBS and vice versa.

TBS was administered using a figure-of-eight magnetic coil with an outer diameter of 70 mm (Magstim Company Ltd., Whitland, Wales, United Kingdom). The iTBS procedure adopted was similar to the one described in Huang et al. (2005), with a 2 s triplet of gamma frequency pulses (50 Hz) applied at a theta rhythm (5 Hz) repeated every 10 s for 190 s to yield a total of 600 pulses. This kind of stimulation design has been found to potentiate the neural activity not only when applied over the motor cortex (Huang et al., 2005) but also over other brain areas including the DLPFC (Hoy et al., 2015). Moreover, a uniform stimulation intensity of 40% of the maximal machine output was used for all participants. This was informed by the limitation in the stimulator’s maximal TBS intensity. Nevertheless, applying uniform stimulation intensity has been used previously to study the functional roles of different cortical brain areas (Ott et al., 2011; Kiyonaga et al., 2014; Vékony et al., 2018; Ngetich et al., 2021). The MNI coordinates (x = 43, y = 41, and z = 34) for the rDLPFC were similar to those used by Fried et al. (2014), which correspond to the right middle frontal gyrus (Petrides, 2019). These coordinates were chosen based on the successful modulation of spatial WM task performance using lower intensity rTMS over this specific region in the aforementioned study (Fried et al., 2014).

To control for the iTBS effect over the rDLPFC on VSWM, we used vertex as a control site. The iTBS similar to that of the rDLPFC was applied over the vertex of each participant, located at (x = 0, y = 0, and z = 90) coordinates, corresponding to the midpoint between the inion and nasion. Before applying iTBS, we obtained AMT for each participant to ensure that our stimulation was tolerable and safe for all participants. This was done only in the first session of each participant. The AMT was defined as the minimum most intensity over the right primary motor cortex required to elicit visible movement of the left first index finger in 5 ≥ out of 10 probes. During the AMT estimation, the participants were instructed to maintain a steady muscle contraction at 20% of the maximal voluntary contraction. However, some TBS studies have used electromyography (EMG) to determine an individual’s motor threshold, a method that has the advantage of providing a quantitative measure of muscle response (Westin et al., 2014). The participants were also instructed to report any discomfort during the stimulation, but there was no report of discomfort from any of them.

To accurately target the stimulation sites, and continuously monitor the position and the orientation of the coil, we used neuronavigation. This was achieved by first co-registering normalized MNI brain to each of the participant’s T1-weighted structural magnetic resonance imaging (MRI) using the Brainsight frameless stereotaxic neuronavigation system (Rogue Research, Montreal, QC, Canada). The T1- weighted images were acquired using a 3.0-Tesla GE Sigma scanner with an 8-channel head coil. During the iTBS, the figure-of-eight coil was placed on the specific site over individuals’ scalp.

### Visuospatial Working Memory Task

**FIGURE 2:**
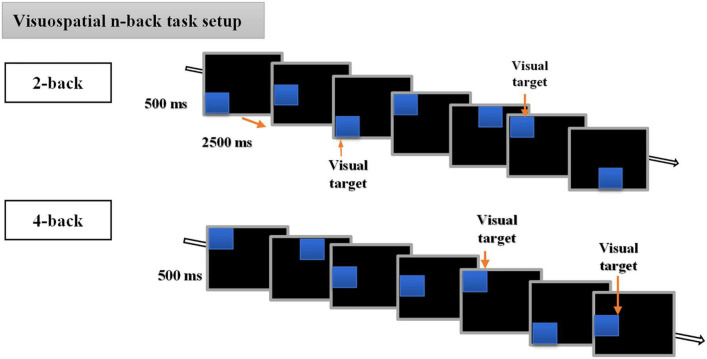
Description of the visuospatial n-back task. The behavioral n-back task consisted of 2-back and 4-back tasks.

In the present study, we used a visuospatial n-back paradigm previously used in our recent cTBS study (Ngetich et al., 2021) and initially modified from the original version of Carlson et al. (1998). The experimental task consisted of a run of 6 blocks, with 3 blocks each for 2-back and 4-back tasks. Each block had (20+n) trials with six visual targets. The participants were required to respond with a keypress when the position of one of the presented stimuli matched that of the previous nth position presented in the sequence. That is two positions or four positions back for 2-back and 4-back tasks, respectively. For the matched stimuli, the participants were instructed to respond by pressing a key “2” on the numeric keypad of a standard keyboard, and not to react if there was no match. At the beginning of every block, the participants were informed whether the current task is a 2-back or 4-back task. Each session lasted for approximately 10 min and the order of n-values for the task was different for each session, i.e., session one could be 4-2-4-2-4-2 and session two could be 2-4-2-4-2-4 but was the same for each participant. The visuospatial n-back task involved the presentation of blues squares on a black background computer screen with a resolution of 1,024 × 768 and a refresh rate of 60 Hz, at eight different random positions (top, bottom, left, right, top left, top right, bottom left, bottom right, bottom right of the central cross), with each trial lasting for 3 s (stimulus duration of 500 ms, and stimulus interval of 2,500 ms) (for detailed illustration, see Figure 2).

### Statistical Analyses

The statistical analyses were performed using IBM SPSS 23.0 software. Each Individual’s accuracy (ACC) scores and reaction times (RTs) were used to evaluate VSWM performance. Based on findings of the previous WM study (Hoy et al., 2015), and those of a study on the effects of iTBS over the primary motor cortex on motor evoked potentials (MEPs) (Huang et al., 2005), we anticipated that iTBS over the rDLPFC would enhance VSWM performance, relative to vertex stimulation and the baseline. To assess whether the iTBS over rDLPFC affected VSWM performance relative to vertex stimulation, and the baseline (pre-stimulation), we analyzed the participants’ accuracy and the reaction times (RTs).

First, to assess accuracy performance, a 2× 3 within-subject repeated measure ANOVA (RM-ANOVA), with load (2back vs. 4-back) and stimulation condition (BL vs. Vertex vs. rDLPFC) as within-subject factors. The accuracy was measured in terms of discriminability index, d prime (d’). The d’ scores were computed from the hit rates (H) and false alarm (FA) rates using the formula: *d*′ = *Z*(*H*)−*Z*(*FA*), where Z represents the transformation of the two distributions, and therefore, makes it possible to differentiate measures with dissimilar ranges of the absolute values (Haatveit et al., 2010). It should be noted that d’ is an effective and efficient measure of WM performance as it is independent of the response bias (John Irwin et al., 2001).

Subsequently, we evaluated the iTBS effect on the accuracy. The iTBS effect on accuracy performance was considered as the difference between the post-stimulation (rDLPFC and vertex), and the pre-stimulation (baseline) d’ scores. Therefore, we subtracted an individual’s mean baseline from the post-stimulation mean d’ scores to get the net effect of iTBS on accuracy (δd’ scores). Thereafter, we conducted a 2×2 RM-ANOVA with Load (2-back vs. 4-back) and site (vertex vs. rDLPFC) as within-subject factors.

We also conducted a 2 (group: sub vs. supra) * 2 (stimulation: DLPFC vs. vertex) *2 (load: 2 vs. 4-back) ANOVA to ascertain whether the application of lower stimulation (subthreshold) affected VSWM performance differently compared to higher stimulation (suprathreshold). What constitutes sub and suprathreshold in TBS remains indeterminate. While most studies have shown that 80% of AMT is sufficient to modulate cognitive performance when applied over implicated brain areas (Ko et al., 2008; Cho et al., 2010; Ott et al., 2011; Li et al., 2014; Hoy et al., 2015; Mcneill et al., 2018; Pestalozzi et al., 2020), one recent study exploring the efficacy of sub and suprathreshold stimulation in the treatment of Major Depressive Disorder used iTBS of 80% AMT as subthreshold, and 120% AMT as suprathreshold (Lee et al., 2021). However, in the present analysis, we considered stimulation < 80% of AMT as subthreshold, and that > 80% AMT as suprathreshold. Therefore, 10 of the participants were categorized into the subthreshold group and 14 into the suprathreshold group.

Following the accuracy analyses, we compared the individual’s RTs under different loads (2-back vs. 4-back), and stimulation conditions (RTs at BL, and after iTBS over the vertex, and the rDLPFC). Similar to the ACC analysis, we conducted a 2×3 within-subject RM-ANOVA, with load (2-back vs. 4-back) and stimulation condition (BL vs. vertex vs. rDLPFC) being the within-subject factors. Only the RTs of the correct responses were included in our analysis. We also conducted a correlation analysis to assess whether the BL performance predicted the stimulation effect, both on the d’ and the mean RTs. In this analysis, the stimulation effect was considered as the difference between the performance after the rDLPFC and the vertex iTBS (i.e., d’(rDLPFC)—d’ (vertex) for d’ and mean RTs (rDLPFC)—mean RTs (vertex).

Finally, *post-hoc* paired *t*-test analyses for ACC and the RTs were conducted to identify the source of significant effects (both main and interaction effects). In addition, paired sample *t*-test was conducted to evaluate the effect of counterbalancing on performance. Importantly, Bonferroni correction was applied for multiple comparisons.

## Results

### Accuracy Performance

Firstly, we performed a two-way repeated measure ANOVA for d’ scores to assess the WM performance under different loads and stimulation conditions. As expected, we found a significant main effect of load [*F*_(1, 23)_ = 93.468, *p* < 0.0001,$\eta_{p}^{2}$ = 0.803], and a significant main effect of stimulation condition [*F*_(2, 22)_ = 10.641, *p* < 0.001,$\eta_{p}^{2}$ = 0.492]. This suggests that VSWM performance was modulated by both the cognitive load (i.e., 2-back and 4-back), and the stimulation condition (i.e., BL and iTBS over either rDLPFC or vertex). Besides, there was also a significant interaction effect between load and stimulation condition [*F*_(2, 22)_ = 3.84, *p* = 0.048,$\eta_{p}^{2}$ = 0.241].

**FIGURE 3:**
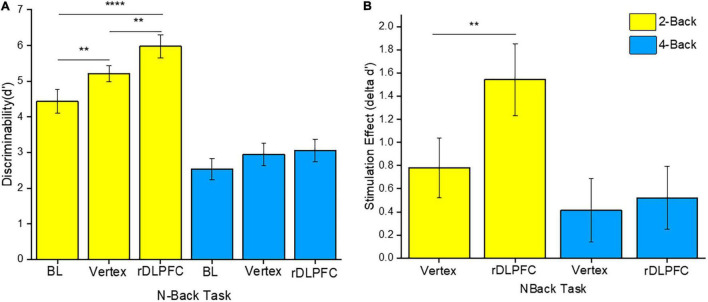
**(A)** Discriminability index for both 2-back and 4-back tasks under different stimulation conditions, namely: Baseline (BL), iTBS over the vertex, and iTBS over the rDLPFC. **(B)** The stimulation effect (δd’ scores). The stimulation effect was obtained by subtracting the BL scores from the post-stimulation scores. The yellow and the blue colors refer to the 2-back and 4-back tasks, respectively. The asterisks indicate the level of significance, with ** indicating *p* ≤ 0.01, and **** indicating *p* ≤ 0.0001. The errors bars indicate the standard mean error (SME).

To determine the source of the effects, we performed a 2-tailed paired *t*-test *post-hoc* test. Since we were mainly interested in the effects of iTBS stimulation on VSWM performance, we compared the same load performances between different stimulation conditions (i.e., at BL, and after iTBS over the rDLPFC or the vertex control). Interestingly, VSWM performance was only enhanced in 2-back, and only after iTBS over the rDLPFC. In particular, the 2-back task performance after iTBS over the rDLPFC was significantly better than that at BL [*t*_(23)_ = 4.961, *p* < 0.0001], and after iTBS over the vertex [*t*(23) = −2.809, *p* = 0.01] as illustrated in Figure 3A. Unexpectedly, the 2-back performance after the iTBS of the vertex was also significantly better than the BL [*t*_(23)_ = −3.046, *p* = 0.006]. This indicates a possibility of practice effects. However, there was no statistically significant difference in performance in the 4-back task between the BL, and after the stimulation over the rDLPFC [*t*_(23)_ = −1.901, *p* = 0.07], or the vertex [*t*_(23)_ = −1.502, *p* = 0.147]. Similarly, the 4-back task performance did not vary after either the stimulation over the rDLPFC or the vertex [*t*_(23)_ = −0.447, *p* = 0.659]. The lack of improvement in task performance in 4-back may reflect the complexity of non-invasively modulating higher load tasks.

Furthermore, to evaluate the actual effect of the stimulation, we conducted a two-way RM-ANOVA for δd’ scores. The δd’ scores were obtained by subtracting the BL d’ scores from the post-stimulation scores (i.e., d’ scores following iTBS over the rDLPFC or the vertex). This was done for both 2-back and 4-back tasks. Our analysis indicated a significant main effect of load [*F*_(1, 23)_ = 4.216, *p* = 0.05,$\eta_{p}^{2}$ = 0.155], and stimulation site [*F*_(1, 23)_ = 5.652, *p* = 0.026,$\eta_{p}^{2}$ = 0.197]. However, there was no significant interaction effect between load and site [*F*_(1, 23)_ = 3.192, *p* = 0.087,$\eta_{p}^{2}$ = 0.122]. Further *post-hoc* analyses showed that the stimulation over the rDLPFC caused a significantly larger effect on 2-back task performance as compared to that over the vertex [*t*_(23)_ = −2.809, *p* = 0.01] as shown in Figure 3B. This suggests a causal role of the rDLPFC in VSWM. Conversely, there was no significant difference between the stimulation effect on d’ scores after the iTBS over the rDLPFC and the vertex in the 4-back task [*t*_(23)_ = −0.45, *p* = 0.66]. Therefore, it may be deduced that the stimulation did not significantly modulate the higher load task.

Besides, the mixed ANOVA for the participants who received sub and suprathreshold yielded a significant main effect of load [*F*_(1, 22)_ = 4.927, *p* = 0.037, $\eta_{p}^{2}$ = 0.183] and a main effect of stimulation [*F*_(1,22)_ = 4.902, *p* = 0.037, $\eta_{p}^{2}$ = 0.182]. There was no other significant main effect or interaction. *Post hoc* analysis showed a group difference, with better performance in 2-back task following iTBS over vertex in subthreshold group as compared to the suprathreshold group [*t*_(22)_ = 3.530, *p* = 0.002]. There were no other group differences.

Additionally, we conducted a paired sample *t*-test analysis to evaluate the effect of counterbalancing. Our analysis did not find significant difference in d’ between the participants who received the iTBS over the right DLPFC in the two separate sessions, either in 2-back [*t*_(11)_ = 0.066, *p* = 0.949] or 4-back [*t*_(11)_ = 0.008, *p* = 0.994]. There was also no significant difference in d’ between those who were stimulated over the vertex in separate sessions, both in 2-back [*t*_(11)_ = 0.580, *p* = 0.573] and 4-back [*t*_(11)_ = 0.855, *p* = 0.411].

### Reaction Time

We conducted a two-way RM-ANOVA to evaluate the RT performance under different WM loads and stimulation conditions. Our analysis found a significant main effect of load [*F*_(1, 23)_ = 27.532, *p* < 0.0001,$\eta_{p}^{2}$ = 0.545]. There was also a significant main effect of stimulation condition [*F*_(2, 22)_ = 6.215, *p* = 0.007,$\eta_{p}^{2}$ = 0.361]. However, there was no significant interaction effect [*F*_(2, 22)_ = 0.073, *p* = 0.93,$\eta_{p}^{2}$ = 0.007].

**FIGURE 4:**
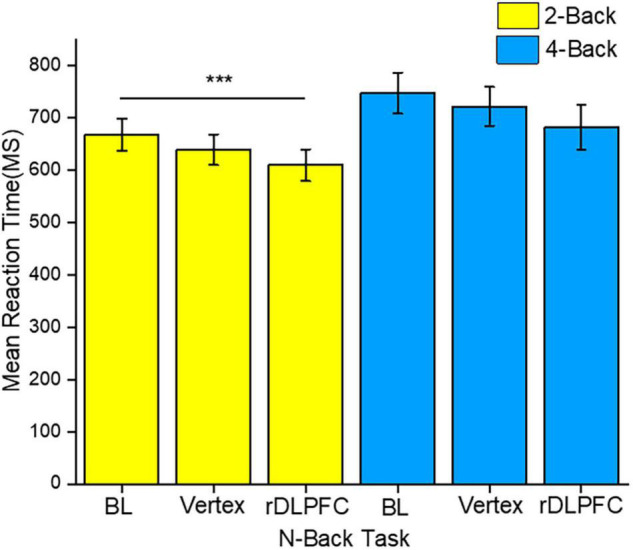
The reaction time (RT) performance for both 2-back and 4-back tasks. The yellow and blue colors indicate 2-back and 4-back tasks, respectively. The asterisks indicate the level of significance, with *** indicating *p* ≤ 0.001. The errors bars indicate the standard mean error (SME).

To identify the source of the main effects, we performed 2-tailed paired *t*-tests. Similar to the accuracy analysis, we compared the same load n-back task RTs between different stimulation conditions. The *post-hoc* tests indicated that the response speed was significantly faster in the 2-back task after iTBS over the rDLPFC as compared to that at the BL [*t*_(23)_ = 3.768, *p* < 0.001]. Other 2-back tasks mean RTs comparisons did not reach significance, with no significant difference between the RTs at BL and after vertex stimulation [*t*_(23)_ = 1.78, *p* = 0.85], nor between the performance after the stimulation over either vertex or DLPFC [*t*_(23)_ = 1.948, *p* = 0.64]. Moreover, there was no statistically significant difference between the 4-back mean RTs across all the stimulation conditions. The RTs at BL were not significantly different from those after the rDLPFC [*t*_(23)_ = 1.942, *p* = 0.061] and vertex [*t*_(23)_ = 1.121, *p* = 0.274]. While there was also no significant difference following the stimulation over either the vertex or the rDLPFC [*t*_(23)_ = 1.443, *p* = 0.163]. Figure 4 clearly illustrates the mean RTs performance.

Finally, we performed a Pearson correlation for baseline performance and the effects of iTBS. However, none of the correlations was significant. The Pearson correlation indicated a lack of significant positive association between the baseline and the post-stimulation d’ both in 2-back (*r* = 0.133, *p* = 0.535) and 4-back (*r* = 0.027, *p* = 0.90) tasks. Also, there was no positive association between baseline and post-stimulation performance RT, both in 2-back (*r* = 0.068, *p* = 0.751) and 4-back (*r* = 0.092, *p* = 0.668) tasks. Nevertheless, a recent study has shown that BL performance level together with pre-stimulation brain state may influence the behavioral impact of TMS (Silvanto et al., 2018). Therefore, TMS studies should evaluate this factor to clearly understand the TMS effect over a targeted brain area on human behavior.

## Discussion

In the present study, we investigated the causal role of the rDLPFC on VSWM performance. To achieve this, we applied iTBS over the rDLPFC, and the vertex (control site). The impact of iTBS on VSWM was evaluated by comparing the n-back task performance at the BL with that after the stimulation over either the rDLPFC or the vertex. Notably, the VSWM task consisted of medium (2-back) and higher load (4-back) visuospatial n-back tasks. Our results indicate that iTBS over the rDLPFC improved visuospatial task performance in 2-back, but not in 4-back tasks. Specifically, iTBS over this target brain area was associated with increased accuracy performance in the medium load task as compared to the performance both at the BL and after the stimulation over the vertex control. However, task performance after the stimulation over the vertex was also significantly better than the BL performance. Indicating a potential practice effect on the accuracy performance. Besides, iTBS over the rDLPFC improved the RT in the 2-back task relative to the BL RT. This notwithstanding, the higher load task performance (both accuracy and RT) was not significantly impacted by the iTBS.

The enhancement of VSWM following iTBS over the rDLPFC was expected, given the importance of DLPFC in WM and the effectiveness of TBS in neuromodulation. Indeed, several studies have established that DLPFC plays a key role in the WM processes (Hoy et al., 2015; Schicktanz et al., 2015; Chung et al., 2018b; Ngetich et al., 2021). In our recent study, we applied cTBS over the same brain area as the present study and observed impairment in the visuospatial 2-back task, which indicates a contrasting effect of cTBS and iTBS when applied over the rDLPFC. Besides, a recent study by Hoy et al. (2015) applied iTBS over the left DLPFC and administered a verbal WM n-back task to assess the stimulation over this area on the WM performance. They found a significantly improved performance in the 2-back task and not in the 3-back task, after the iTBS over the left DLPFC. More importantly, the aforementioned study found that iTBS was associated with an increase in the frontoparietal connectivity, and more so, prominent parietal gamma power relative to the sham stimulation (Hoy et al., 2015).

Furthermore, a recent study investigating inter-and intra-individual iTBS variability, indicates that iTBS induces robust and relatively consistent cortical modulation effects within and between individuals (Hinder et al., 2014). Perhaps this makes it suitable for both research and clinical application. While recent review and meta-analytic studies alongside the earlier reviewed studies indicate that non-invasive brain stimulation over the DLPFC influences WM task performance (Brunoni and Vanderhasselt, 2014; Lowe et al., 2018; Widhalm and Rose, 2019). It is, therefore, interesting to observe that iTBS over the rDLPFC in the present study led to enhancement in VSWM performance. As mentioned earlier, there is evidence suggesting hemispheric specialization in the processing of verbal and visuospatial information. In particular, the right hemisphere has been reported to be involved in the processing of the visuospatial content (Jonides et al., 1993; Kessels et al., 2000), while the processing of the verbal information has been primarily linked to the left hemisphere (Fried et al., 2014). Therefore, the reported effect in our study is in line with the previous studies and supports the role of the right hemisphere, especially the right DLPFC in VSWM.

Moreover, it should be noted that the strength of the frontoparietal network has been positively correlated with the WM performance both in healthy participants (Nee and Brown, 2013) and patients (Figueroa-Vargas et al., 2020). While high gamma power over the peak but not the trough has been found to boost memory performance (Alekseichuk et al., 2016). Despite not collecting electrophysiological or imaging data, it is deducible from the previous studies that perhaps, iTBS over the rDLPFC significantly improved the frontoparietal connectivity and thus enhanced VSWM performance.

The lack of a significant performance enhancement in the 4-back task in the present study can be attributed to some key factors. Firstly, it is likely that the participants were already performing close to or at their highest possible levels. This does not necessarily imply that the VSWM performance itself was already near the maximum possible levels, but perhaps the participants’ abilities to perform the higher load task were already stretched to the limits. Therefore, despite the facilitatory effect of iTBS, the significant enhancement in cognitive processing could not modulate the “ceiling performance”. Importantly, the extent of facilitation associated with the stimulation, especially in healthy participants, cannot surpass an individual’s natural potential (Hoy et al., 2015). Secondly, it is possible that since the cognitive processing resources are directed naturally toward a relatively complex high-load task, iTBS may not significantly potentiate its already optimized performance. Interestingly, high load task is associated with the deactivation of the default mode network (DMN) (Mckiernan et al., 2003; Thomason et al., 2009), increased activation, particularly in the key brain areas such as the frontoparietal (Tomasi et al., 2008), and generally decreased distractibility (Sörqvist et al., 2016). Therefore, it is expected that the aforementioned occurrences may facilitate the reduction of error rate and the realization of the optimal level performance that may not be significantly improved further by the iTBS.

Additionally, the observed iTBS effect only in 2-back and the lack of it in 4-back tasks may also be explained by a phenomenon called stochastic resonance. This phenomenon is characterized by beneficial effects of unpredictable fluctuations such as facilitation of the response to a weak signal by random noise (Stocks, 2000; McDonnell and Abbott, 2009; Romei et al., 2016). Stochastic resonance has been intensely studied and quantified in several physical and biological systems such as neurons (Stocks, 2000; McDonnell and Abbott, 2009). Recently, a study by Silvanto and Cattaneo (2017) established that varying TMS intensities affect neural firing differently. In particular, the aforementioned study suggests that low-intensity TMS enhances early neural firing while higher intensity suppresses it (Silvanto and Cattaneo, 2017). Considering that the AMT varied from one participant to another in the present study, it is likely that administering iTBS at a uniform intensity of 40% of the MSO for all participants might have led to a situation where some participants received sub or suprathreshold stimulation (corresponding to individual’s AMT). And since an earlier study (Silvanto et al., 2007) found that TMS reactivated WM for weak representations, it is possible that the subthreshold stimulation preferentially improved the performance in the lower load VSWM tasks (2-back). In particular, despite our analysis finding better performance in 2-back only following vertex stimulation in subthreshold compared to a suprathreshold group, such finding is an important indicator of the possible stochastic resonance effect, as there were no observable significant differences in 4-back tasks between the two groups. The significant main effect of group is also an important pointer to how performance can be affected differently by sub and suprathreshold stimulation intensities. Therefore, future iTBS studies should use a larger sample size to elucidate the potential impact of this phenomenon on cognitive task performance.

More importantly, our findings demonstrate that iTBS enhances behavioral performance, thus adding to the critical evidence suggesting that the modulatory effects of iTBS extend beyond the motor cortex (Hoy et al., 2015; Chung et al., 2018b). This is especially interesting since our recent study found that cTBS over the rDLPFC impairs VSWM performance (Ngetich et al., 2021), which is opposite to the results of the present study. Also, other studies have consistently found an impairment of verbal WM performance by cTBS of the DLPFC (Schicktanz et al., 2015; Vékony et al., 2018; Ngetich et al., 2021). As we discussed earlier, the original study by Huang et al. (2005) found that iTBS over the primary motor cortex significantly increased motor evoked potentials (MEPs), while cTBS over the same brain area decreased the MEPs.

Therefore, does it necessarily mean that iTBS over the neural cortex enhances cognitive performance while the cTBS decreases it? The evidence suggests otherwise. Apart, from individuals’ factors such as age, sex, and endogenous brain oscillations (Ridding and Ziemann, 2010), other specific factors such as (1) the cognitive task, and (2) the functional role of the targeted brain area, influence the direction of behavioral effects associated with TBS (Ngetich et al., 2020). Whereas iTBS generally facilitates neural activity, while cTBS inhibits it (Huang et al., 2005, 2011; Li et al., 2019), the behavioral outcome may vary accordingly. For instance, Kaller et al. (2011) found a functional dissociation between the right and the left DLPFC in planning. In particular, cTBS over the left DLPFC resulted in global acceleration, while that of the right led to global deceleration of the planning processes (Kaller et al., 2011). Therefore, it may be argued that the inhibition of the neural activity of the left DLPFC led to suppression of other competing cognitive processes, and thus enhancement of the cognitive performance. This phenomenon has been termed as addition by subtraction (Luber and Lisanby, 2014). Such behavioral effects that are negatively correlated with the size of the neural activity pose an interesting challenge to the clinical application of the TBS. In essence, it is imperative to understand the cognitive deficiencies associated with specific mental health conditions, and specific neurophysiological modulation occasioned by a particular psychiatric condition. For instance, the hyperactivity and hypoactivity of the right and left DLPFC, respectively, in medication-resistant depression necessitates the application of cTBS over the rDLPFC and iTBS to the left when using a combined cTBS + iTBS treatment protocol (Li et al., 2014). Similarly, findings regarding the brain areas significantly involved in VSWM could be beneficial in the treatment of psychiatric conditions characterized by the deficiency of this cognitive process, like schizophrenia (Cocchi et al., 2009).

Nevertheless, the possible influence of the practice effects in the present study makes it necessary to interpret our findings with caution. Despite our attempt to limit practice effects by using different series of VSWM n-back tasks for different sessions, it still exerted its influence on the performance. Although the accuracy performance in the 2-back task was significantly better after iTBS over the rDLPFC than that at the BL and following iTBS of the vertex, the fact that the performance after vertex stimulation was significantly better than the BL performance, suggests that the practice effects influenced VSWM performance. Interestingly, in our previous cTBS study, the practice effects were not apparent (Ngetich et al., 2021), suggesting that while cTBS may suppress practice effects (Vékony et al., 2018), iTBS may not significantly modulate them. However, the influence of practice effects on cognitive task performance is not generally unexpected. One recent study has shown that a repeated practice with a specific task, even when using different sets of stimuli necessarily results in an enhancement of the subsequent task performance (Dutilh et al., 2011). Furthermore, other related studies have reported the possible influence of practice effects on cognitive task performance (Hoy et al., 2015; Vékony et al., 2018). This notwithstanding, the apparent practice effects did not entirely affect the observation of the impact of iTBS on VSWM.

Moreover, the BL performance was only assessed in the second session, and thus it may not be possible to ascertain the level of BL performance in the third session. Therefore, future studies should consider evaluating BL performance in all stimulation sessions to measure the stimulation effect and to balance the number of tasks across the different sessions. Also, the stimulation was applied at a uniform stimulation intensity of 40% of the MSO. Therefore, since motor thresholds may vary from one individual to another, the stimulation intensity should be adapted to individuals’ MTs to ensure uniform stimulation for all participants. Finally, the other limitation of the present study lies in the use of only behavioral tests. This is despite the previous studies including (Polanía et al., 2018) suggesting that it is possible to integrate non-invasive brain stimulation (NIBS) with other techniques such as EEG and fMRI. Thus, future related studies should integrate TBS with EEG or fMRI to determine the most crucial frequency to target and electrophysiological effects of TBS, and to assess the functional connectivity associated with VSWM and the modulation of such connectivity by TBS, respectively. Notably, Romei et al. (2016) suggest that NIBS can be enhanced through rhythmic TMS, which target endogenous neural oscillations via entrainment or phase cancelation.

In conclusion, we have demonstrated that iTBS over the rDLPFC improves VSWM performance. Our findings suggest that the aforementioned brain area plays an important role in VSWM, and that iTBS is a safe and effective technique for investigating the causal role of the specific brain areas. Overall, the present study contributes to the understanding of the modulatory effects of TBS and may have a clinical application, especially in the modeling of the brain stimulation treatment intervention for neuropsychiatric conditions associated with the deficits in the VSWM.

## Data Availability Statement

The raw data supporting the conclusions of this article will be made available by the authors, without undue reservation.

## Ethics Statement

The studies involving human participants were reviewed and approved by the Committee for the Protection of Human Subjects of the University of Electronic Science and Technology of China (UESTC). The patients/participants provided their written informed consent to participate in this study.

## Author Contributions

RN, JZ, ZJ, and LL conceived and designed the experiments. RN, DJ, and BS performed the experiments. RN and WL analyzed the data. RN wrote the manuscript. All authors reviewed the manuscript.

## Conflict of Interest

The authors declare that the research was conducted in the absence of any commercial or financial relationships that could be construed as a potential conflict of interest.

## Publisher’s Note

All claims expressed in this article are solely those of the authors and do not necessarily represent those of their affiliated organizations, or those of the publisher, the editors and the reviewers. Any product that may be evaluated in this article, or claim that may be made by its manufacturer, is not guaranteed or endorsed by the publisher.
